# Antimitochondrial Antibody–Negative Primary Biliary Cholangitis in a Patient with a History of Colon Cancer and Chemotherapy: A Case Report

**DOI:** 10.1055/s-0046-1819571

**Published:** 2026-04-16

**Authors:** Mohammad Berro, Kenana Tawashi, Yahya Ibrahim, Mohammad Sharaf, Somar Ahmad, Ayman Ali

**Affiliations:** 1 Faculty of Medicine, Damascus University, Damascus, Syria; 2 Department of Oncology, Al Biruni University Hospital, National University Hospital, Damascus, Syria; 3 Gastroenterology Department, National University Hospital, Damascus University, Damascus, Syria

**Keywords:** primary biliary cholangitis, antimitochondrial antibodies, ursodeoxycholic acid, liver disease

## Abstract

**Background:**

Primary biliary cholangitis (PBC) is a chronic, immune-mediated liver disease with progressive destruction of bile ducts, cirrhosis, and liver failure. Diagnosis is usually based on detecting antimitochondrial antibodies (AMA), found in 95% of patients with PBC. However, some patients test negative for AMA and other antibodies, which complicates the diagnosis process.

**Case Presentation:**

We report a case of a 57-year-old Syrian woman who presented with fatigue, jaundice, pruritus, and abdominal pain, and her medical history included colon cancer and chemotherapy. Laboratory tests showed liver fibrosis but were negative for AMA, antinuclear antibodies, and anti-smooth muscle antibodies, further complicating the diagnosis. A liver biopsy was performed and showed mixed fibrosis, cholangitis, and cholestasis, which led to the diagnosis of AMA-negative PBC.

**Conclusions:**

This case confirms the importance of liver biopsy in unclear cases of primary cholangitis. This case also highlights the patient's history of colon cancer and chemotherapy as a notable clinical observation, generating a hypothesis for a potential association that warrants further systematic investigation. A better understanding of such variants in PBC can enable early diagnosis and improve management strategies for affected patients.

## Introduction

Primary biliary cholangitis (PBC) is a chronic, immune-mediated liver disease with a progressive course. Genetic and environmental factors are believed to contribute to its onset. The disease primarily affects women over 50. 1 Incidence and prevalence rates vary widely (figures across regions are 1.76 and 14.60 per 100,000 persons, respectively), with the Asia-Pacific region reporting the lowest figures. 2 The two main symptoms are fatigue and pruritus, although there is no strong correlation between these symptoms and the disease stage. Diagnosis involves a series of serological tests, mainly the antimitochondrial antibody (AMA) test. A liver biopsy is often unnecessary but can be used to confirm the diagnosis. 3 AMA is found in approximately 95% of PBC patients, with an AMA test sensitivity of 84% (95% confidence interval [CI] = 77–90%) and a specificity of 98% (95% CI = 96–99%). 4 PBC treatment is typically based on the use of ursodeoxycholic acid (UDCA), which can reduce the need for liver transplantation and slow disease progression. UDCA appears to have a similar effect in both AMA-positive and AMA-negative patients. Obeticholic acid (OCA) can also be used in combination with UDCA or as monotherapy. Patients with decompensated cirrhosis should be evaluated for liver transplantation. It is important to note that the symptoms do not tend to improve with UDCA or OCA treatment. Management of symptoms is important in order to improve quality of life. 3

We report a case of a symptomatic PBC patient who tested negative for AMA and other well-known antibodies. Their absence made the diagnosis very difficult, especially since PBC is largely considered an immune-mediated disease.

## Case Report

A 57-year-old Syrian woman was referred to our department, complaining of fatigue and scleral icterus. These symptoms started 7 days ago, accompanied by mild, persistent abdominal pain in the right hypochondrium, which worsened after eating and resolved after defecation. The patient also reported fever (unmeasured), pruritus, and dark urine over the preceding 2 days.

Her medical, surgical, and drug history revealed that she had undergone a colon tumor resection followed by seven courses of chemotherapy over 18 months. The chemotherapeutic regimen consisted of 5-fluorouracil with oxaliplatin, administered in seven cycles, with the last treatment occurring a year ago. Her psychosocial history indicated a smoking history of 20 pack-years. Her familial and allergic history was unremarkable. The patient's vital signs were as follows: blood pressure (90/50 mmHg), heart rate (110 bpm), with respiratory rate, temperature, and oxygen saturation within normal limits. Physical examination showed that the patient was conscious, responsive, and oriented to time, place, and person; she presented with jaundice, dyspnea (grade III), dysuria, polyuria, and dark urine. Abdominal examination revealed tenderness in the epigastrium and right hypochondrium, with positive right upper quadrant tenderness. Other systems were within normal limits. Laboratory values are detailed in Table 1 . Urinalysis showed clear orange urine with a specific gravity of 1.025, pH 5, bilirubin (++), 5 white blood cells, and 5 red blood cells per high-power field. Urine culture was negative. Abdominal ultrasound and computed tomography (CT) were unremarkable.

**Table 1:** Laboratory values on admission

Complete blood count (CBC)		
**Test**	**Result**	**Reference range**
Hemoglobin (Hb)	13.3 g/dL	12.0–16.0
Hematocrit (Hct)	41.5%	36–46
RBC	4.7 × 10 ^6^ /µL	4.0–5.5
WBC	9.4 × 10 ^3^ /µL	4.0–11.0
Neutrophils/lymphocytes	66%/31%	40–75/20–45
Platelets	189 × 10 ^3^ /µL	150–400
MCV	88 fL	80–100
MCH	28 pg	27–33
**Biochemistry and liver function tests**		
**Test**	**Result**	**Reference range**
ALT	265 U/L	7–56
AST	217 U/L	10–40
ALP	218 U/L	44–147
LDH	259 U/L	140–280
Total bilirubin	15.04 mg/dL	0.3–1.2
Direct bilirubin	11.99 mg/dL	0.0–0.3
Albumin	3.4 g/dL	3.5–5.0
Total protein	5.6 g/dL	6.0–8.3
CPK	24 U/L	38–174
**Renal function and metabolic tests**		
**Test**	**Result**	**Reference range**
Urea	57 mg/dL	15–45
Creatinine	0.4 mg/dL	0.6–1.3
Uric acid	2.7 mg/dL	3.5–7.2
Glucose (random)	99 mg/dL	70–140
**Electrolytes and minerals**		
**Test**	**Result**	**Reference range**
Sodium	135 mmol/L	135–145
Potassium	4.0 mmol/L	3.5–5.1
Chloride	101 mmol/L	98–107
Phosphorus	3.2 mg/dL	2.5–4.5
Calcium	9.2 mg/dL	8.6–10.2
**Coagulation and inflammatory markers**		
**Test**	**Result**	**Reference range**
PT ratio	22.5%	70–120
INR	1.77	0.8–1.2
PTT	39.8 s	25–35
CRP	11.4 mg/L	<6
**Iron studies and vitamins**		
**Test**	**Result**	**Reference range**
Serum iron	54 µg/dL	60–170
TIBC	240 µg/dL	250–370
Transferrin	240 mg/dL	200–360
Vitamin B12	2000 pg/mL	200–900
**Serology and autoimmune markers**		
**Test**	**Result**	
Wright test	Negative	
Widal test	Negative	
HBsAg	Negative	
Anti-HCV Ab	Negative	
Anti-HAV Ab	Negative	
AMA-M2	Negative	
ASMA	Negative	
ANA	Negative	

Abbreviations: ALP, alkaline phosphatase; ALT, alanine aminotransferase; ANA, antinuclear antibody; ASMA, anti-smooth muscle antibody; AST, aspartate aminotransferase; CRP, C-reactive protein; INR, international normalized ratio; LDH, lactate dehydrogenase; MCH, mean corpuscular hemoglobin; MCV, mean corpuscular volume; PT, prothrombin time; PTT, partial prothrombin time; RBC, red blood cell; TIBC, total iron-binding capacity; WBC, white blood cell.

During her admission (approximately 20 days), the patient's clinical status and laboratory tests deteriorated. She remained conscious and responsive but became disoriented to time and place, and her jaundice worsened. Relevant laboratory results are shown in Table 2 . Serological testing was negative for AMA, specifically tested via AMA-M2 ELISA, as well as for antinuclear antibodies (ANA) and anti-smooth muscle antibodies (ASMA; Table 1 ). Therefore, a liver biopsy was indicated. Several differential diagnoses were considered, such as PBC, primary sclerosing cholangitis (PSC), and—most suspected—micrometastases from the resected colon tumor. The biopsy was performed after a cardiovascular consultation, with prothrombin time, international normalized ratio, and partial thromboplastin time improved via plasma transfusion.

**Table 2:** Laboratory values after 20 days of admission

Complete blood count		
**Test**	**Result**	**Reference range**
Hemoglobin	9.0 g/dL	12.0–16.0
Hematocrit	28%	36–46
RBC	2.96 × 10 ^6^ /µL	4.0–5.5
Platelets	96 × 10 ^3^ /µL	150–400
**Liver function and coagulation**		
**Test**	**Result**	**Reference range**
Albumin	3.5 g/dL	3.5–5.0
Total bilirubin	22.39 mg/dL	0.3–1.2
Direct bilirubin	12.41 mg/dL	0.0–0.3
PT ratio	33%	70–120
INR	2.3	0.8–1.2
PTT	38 s	25–35

Abbreviations: INR, international normalized ratio; PT, prothrombin time; PTT, partial prothrombin time; RBC, red blood cell.

The histopathological examination revealed mixed macronodular and micronodular cirrhosis with moderate activity (grade 3, stage 4); fibrosis of liver tissue cores with well-formed, variably sized nodules; inflammatory infiltration, including neutrophils, with no significant plasmacytic cells; proliferation of bile ductules with bile duct inflammation (cholangitis); mild to moderate cholestasis; no granulomatous formation or neoplastic changes. The histologic findings were suggestive of PBC.

Based on laboratory and clinical findings at day 20, the patient's Child–Pugh score was calculated as 15 (Class C), and her model for end-stage liver disease (MELD) score was 18, consistent with advanced liver disease and high priority for transplantation. We explained that liver transplantation was the best treatment option, but due to the difficult economic situation in our country, the patient and family refused it. The patient was discharged on UDCA (500 mg twice daily) and palliative therapy 2 days after the biopsy. Follow-up 1 month later showed no improvement in her condition.

## Discussion

PBC, formerly known as primary biliary cirrhosis, is a chronic autoimmune disease characterized by the gradual destruction of small bile ducts in the liver, accompanied by the presence of specific immune antibodies and inflammation. 5 PBC can occur without AMA, a condition known as AMA-negative PBC. These patients exhibit clinical and histopathological features of PBC but do not test positive for AMA. 6 Without timely identification and treatment, many patients with PBC face severe outcomes, such as liver failure, the need for a transplant, or even death within a decade. 7 Globally, the incidence and prevalence of PBC are 1.76 and 14.60 per 100,000 people, respectively. However, these rates vary by region. In the Asia-Pacific region, the incidence is lower than in North America and Europe. 2 AMA-negative PBC is a very rare subtype, accounting for only 5 to 10% of all PBC cases. In the absence of histological evidence of autoimmune hepatitis (AIH), ANA and ASMA are often present. 6 However, in our case, both ASMA and ANA were negative. Unfortunately, AMA-negative PBC tends to be more severe and has a worse progression compared to AMA-positive PBC, with a higher risk of liver complications, including an increased need for organ transplantation and increased mortality. 6 8 To diagnose PBC, two out of three main criteria must be met: elevated alkaline phosphatase (ALP) levels, the presence of AMA or other specific autoantibodies such as Sp100 or gp210 if AMA is negative, and histologic evidence. 3 Based on this information, our patient was diagnosed with PBC due to elevated ALP levels and histologic findings. Most PBC patients do not need a liver biopsy for diagnosis, as clinical and lab tests are usually sufficient. However, a biopsy is necessary if the diagnosis is unclear or if another condition like AIH or nonalcoholic steatohepatitis is suspected. If a biopsy is done, finding non-suppurative cholangitis and damage to small or medium-sized bile ducts strongly supports a PBC diagnosis. Additionally, if there's a suspicion of overlap syndrome due to AIH features, a biopsy is crucial. 7

The response to UDCA treatment, which is the primary treatment for PBC, is similar for both AMA-negative and AMA-positive PBC patients, but it is still unclear if there are differences in their clinical outcomes. 3 It is usually started gradually and can be given twice or once daily, often at bedtime, to help with compliance. UDCA works through various mechanisms, including promoting bile flow, protecting liver cells, reducing inflammation, and modulating the immune system. 9 Studies have shown that UDCA can reduce the need for liver transplants in PBC patients. 10 UDCA is effective for PBC patients at any stage, but those in the early stages respond better than those with advanced disease. Liver transplant indications for PBC patients are similar to those for other chronic liver diseases. Patients should be evaluated for a transplant if they have decompensated cirrhosis, a MELD score over 15, total bilirubin above 6 mg/dL, or a Mayo risk score over 7.8. About 20 to 30% of PBC patients who undergo transplantation experience disease recurrence within 10 years, and up to 50% within 20 years. 3 Our patient had a Child–Pugh score of 15 and a MELD score of 18, meeting criteria for transplant evaluation.

Due to the lack of liver transplantation availability in Syria, the patient was placed on UDCA treatment. The differential diagnosis initially included PSC and autoimmune overlap syndromes; however, several findings argued against these. PSC is classically diagnosed by the demonstration of irregular multifocal strictures and alternating dilatations (“beading”) of the intrahepatic and extrahepatic bile ducts on cholangiography—typically obtained by magnetic resonance cholangiopancreatography (MRCP) or endoscopic retrograde cholangiopancreatography (ERCP)—which were not present on abdominal imaging in this case, reducing the likelihood of PSC. In PSC, this cholangiographic pattern reflects the characteristic chronic inflammatory and fibrotic process affecting the bile ducts and is a major diagnostic criterion after excluding secondary causes of sclerosing cholangitis. Liver histology in PSC often shows periductal concentric fibrosis and bile duct abnormalities, rather than the histologic pattern observed here. 11 In contrast, the liver biopsy showed features consistent with PBC—nonsuppurative destructive cholangitis with interlobular bile duct damage and cholestatic changes without the pronounced interface hepatitis typical of AIH or a PSC–AIH overlap—which supports the diagnosis of PBC. 12 Common infectious, obstructive, and ischemic causes of cholestasis were excluded by negative viral serologies, unremarkable imaging, and clinical context, and a normal C-reactive protein further argued against an active infectious or severe inflammatory biliary process.

In evaluating this patient, key alternative diagnoses were also considered. Metastatic disease from the patient's known history of colon cancer was deemed unlikely based on the absence of focal lesions on abdominal ultrasound and non-contrast CT imaging, further corroborated by the lack of neoplastic changes on liver histology. Regarding drug-induced liver injury (DILI) related to prior chemotherapy (oxaliplatin/5-FU), the prolonged symptom-free interval of approximately 1 year argued strongly against a classic acute cytotoxic injury pattern, although a delayed or idiosyncratic immune-mediated reaction could not be entirely excluded. It is noted that specific tumor markers (e.g., CEA [carcinoembryonic antigen]) were not assessed in this clinical workup, which limits the ability to definitively rule out metastatic activity.

## Conclusions

This case emphasizes the importance of considering AMA-negative PBC in the differential diagnosis, even when other antibodies such as ASMA and ANA are also negative. Additionally, it highlights the patient's history of colon cancer and chemotherapy as a notable clinical observation, generating a hypothesis for a potential association that warrants further systematic investigation. A liver biopsy should not be delayed in such situations to ensure accurate diagnosis and appropriate treatment.

## Limitations

This case report has several inherent limitations that should be acknowledged. First, the diagnosis was established in a resource-constrained setting. Consequently, several recommended diagnostic modalities were not available, including serological testing for specific antibodies (anti-gp210, anti-Sp100, IgM), advanced cholangiographic imaging (MRCP/ERCP), and tumor markers such as CEA. Second, detailed technical specifications of the liver biopsy (e.g., exact number of cores, stain details) could not be retrieved, and biopsy photomicrographs are unavailable for publication. Third, while the identified 1-year interval makes acute DILI unlikely, a comprehensive long-term follow-up to monitor the response to UDCA therapy was not possible due to loss of contact with the patient. Finally, the potential association with prior chemotherapy remains a clinical observation based on a single case; it generates a hypothesis but cannot establish causality without robust epidemiological support.
